# Evaluation of the Apoptotic, Prooxidative and Therapeutic Effects of Odoroside A on Lung Cancer: An In Vitro Study Extended with In Silico Analyses of Human Lung Cancer Datasets

**DOI:** 10.3390/life15030445

**Published:** 2025-03-12

**Authors:** Fatma Seçer Çelik, Göksemin Fatma Şengül, Safaa Altveş, Canan Eroğlu Güneş

**Affiliations:** 1 Department of Medical Biology and Genetics, Faculty of Medicine, Ankara Medipol University, 06050 Ankara, Turkey; 2 Department of Medical Biochemistry, Faculty of Medicine, Ankara Medipol University, 06050 Ankara, Turkey; goksemin.sengul@gmail.com; 3 Science and Technology Research and Application Center (BITAM), Necmettin Erbakan University, 42005 Konya, Turkey; safaa.e.t@gmail.com; 4 Department of Medical Biology, Faculty of Medicine, Necmettin Erbakan University, 42005 Konya, Turkey; cananeroglu88@gmail.com

**Keywords:** lung cancer, apoptosis, CASP3, redox hemostasis, odoroside A

## Abstract

Objective: The apoptotic effects of odoroside A on lung cancer cells were studied in our project. We also supported and extended our experimentally-proven results via bioinformatics analysis on human lung cancer tissues. Materials and Methods: In vitro studies were conducted using the A549 cell line. Cell proliferation was evaluated through a CCK-8 assay. For gene expression analysis, the qRT-PCR method was used, while CASP3 protein levels were detected using Western blotting and ELISA. Moreover, the oxidant status of cells was determined by measuring TAS and TOS levels. To construct a protein–protein interaction network, STRING analysis was performed. For the determination of differential expression of apoptosis-related genes, the GEPIA tool was utilized. Kaplan–Meier plots with overall survival, disease-specific survival and progression free intervals were obtained from UCSC Xena to evaluate the prognostic value of caspases. Results: The gene expression levels of *CASP3*, *CASP7*, *CASP8*, *CASP9*, *FAS*, and *FADD* were elevated between 4–16 fold in Odo A-treated lung cancer cells compared to controls. CASP3 protein expression was significantly higher in Odo A-treated cancerous cells than the control group. Low TAS (0.5700 ± 0.0067 in Odo A vs. 0.6437 ± 0.0151 in control) and high TOS (0.82800 ± 0.0208 in Odo A vs. 0.6263 ± 0.0258 in control) levels as well as high OSI values (1.4531 ± 0.0414 in Odo A vs. 0.9748 ± 0.0539 in control) were obtained. Correlogram and protein–protein network analyses suggested strong correlations and interactions among target genes. Lastly, Kaplan–Meier analysis showed no prognostic value of caspases, but potential therapeutic targets in lung cancer. Conclusions: Anti-cancer, prooxidative and therapeutic effects of Odo A on lung cancer cells were shown in our study. These data were supported and extended via computational analyses that we performed. In conclusion, Odo A could be used in clinics to treat patients with lung cancer.

## 1. Introduction

Cancer is one of the most common causes of mortality worldwide. In particular, lung cancer (LC) has the highest rate of morbidity and mortality among all prevalent malignancies [1]. LC can be broadly classified into two categories based on the histology of cancer cells: small cell lung cancer (SCLC) and non-small cell lung cancer (NSCLC). Of these types of lung cancer, NSCLC is the most prevalent subtype, making up 85–90% of cases. Large cell LC, lung squamous carcinoma, and lung adenocarcinoma (LUAD) are among several other histological subtypes NSCLC [2].

A class of substances known as cardiac glycosides acts as an allosteric inhibitor of Na+/K+-ATPase [3]. Cardiac glycosides are divided into two groups: cardenolides (a five-membered butyrolactone ring) and bufadienolides (a six-membered pyrone ring). Both groups share common structures, including a steroid core, an unsaturated lactone ring, and a sugar component [4,5]. Using room temperature water to macerate *N. oleander* leaves, these monoglycosidic cardenolides (odoroside A, H, sarmentoside, oleandrin, and oleandrigenin) containing the structure of 3β,14β-dihydroxy-5β-card-20(22)-enolide, with or without an acetoxy group at C-16, were found to show significant anti-cancer activity and could be extracted preferentially (in comparison to less active genins, di- or triglycosides) for therapeutic purposes [6]. Clinical data in a retrospective study conducted by Kepp et al. (2012) revealed that cardiac glycosides, specifically digoxin, prolonged the survival of carcinoma patients treated with chemotherapy. Cardiac glycosides were also proposed to regulate the immune response at multiple levels. It has been found that these agents trigger the immunogenic death of cancer cells, thereby contributing to their clinical anti-cancer activity [7]. *N. oleander* does not only include oleandrin, but also contains additional chemicals, such as odoroside A, nerigoside, and oleandrigenin, contributing to its cytotoxic effect [8,9]. It has already been determined that odoroside A (Odo A) has antitumorigenic activity in gastritis, colon, and cervical cancers [10]. In another study, Odo A was proven to show an anti-metastatic and anti-cancer effect on highly metastatic and radio-resistant breast cancer cells [11]. Moreover, Odo A and its derivatives activated the ROS/JNK pathway to induce autophagy and apoptosis, which in turn suppressed the progression of leukemia. These findings imply that these chemicals might possess antitumorigenic properties against leukemia, particularly acute myeloid leukemia (AML) [12].

An imbalance between the body’s production of free radicals and antioxidant defenses leads to oxidative damage, which is accepted as the hallmark of oxidative stress [13]. When the antioxidant capacity of the living organism is insufficient, an excess of unstable and reactive free radicals interacts with RNA, DNA, lipids, and proteins, resulting in oxidative damage [14]. Cancer initiation and progression cause changes in cellular oxidant levels [15,16]. Triggering apoptosis might be a useful mechanism to eliminate cancer cells. Disturbances in the redox homeostasis in the favor of prooxidant production directs the cancer cells to apoptosis. Therefore, many herbal extracts are frequently used in anti-cancer studies due to their prooxidation properties [17,18].

Our aim in this study is to investigate the in vitro apoptotic effect of Odo A in lung cancer cells, and also to elucidate the molecular connections of the apoptotic genes/proteins using in silico tools.

## 2. Materials and Methods

### 2.1. Cell Culture

The human lung cancer cell line A549 was obtained from the American Type Culture Collection (ATCC, Cat.No CCL-185™, Manassas, VA, USA). Proliferation of the cell line A549 was achieved in the appropriate culture medium using F-12K supplemented with 10% fetal bovine serum, 2 mM L-glutamine, and 1% penicillin–streptomycin. Cell proliferation, passage and follow-up processes were monitored under an inverted microscope. Cultured cells were incubated in 95% humidity with 5% CO_2_ until sufficient growth was achieved.

### 2.2. CCK-8 Cell Proliferation Assay

A 96-well cell culture plate (Corning Inc., Corning, NY, USA) was seeded with roughly 4000 lung cancer cells per well. First, 1 mg of Odo A (Sigma Aldrich, Cat.No S961795, Darmstadt, Germany) was completely dissolved in 200 µL of DMSO in a controlled manner until no single particle was visible in the solution. Then, 10 mL of culture medium was added into the DMSO-dissolved Odo A solution and 192 µM of initial stock solution was prepared. Next, this initial stock solution was diluted to 100 µM dilution stock in a culture medium that was further diluted to 1000 nM by again adding culture medium to prepare the final working stock. Then, a series of Odo A concentrations (ranging from 0–1000 nM), diluted from the final working stock solution, were administered to the culture plates and cells were incubated for 24 and 48 h at 37 °C in a humid environment containing 5% CO_2_. Each measurement was performed in three duplicates. Following the incubation period at 37 °C, 10 μL of the CCK-8 reagent (MedChemExpress, Cat.No HY-K0301, South Brunswick, NJ, USA) was added to each well. After 2 h, the optical density (OD) of each well was measured at 450 nm using a multifunction microplate reader (Infinite M200 Pro, Tecan, Männedorf, Switzerland). Cell viability was calculated for each concentration as a percentage of the mean of the corresponding negative control.

### 2.3. qRT-PCR Analysis

Total RNA from A549 cells was isolated using QIAzol lysis reagent (Qiagen, Cat.No 79306, Hilden, Germany) and 1 mg of cDNA was synthesized, corresponding to 400 µg in 10 µL reaction mix (40 µg/µL), using the Transcriptor First-Strand cDNA Synthesis Kit (Bio-Rad, iScript™ cDNA Synthesis Kit, Cat.No 170-8851, Hercules, CA, USA), both of which were used in accordance with the manufacturer’s instructions. The primer sequences for the apoptotic genes included in the qRT-PCR analysis were designed using IDT PrimerQuest; Table 1 displays the outcomes [19]. The designed primers were blasted on the homo sapiens genome at the blast.ncbi.nlm.nih.gov/Blast website. Only sequences targeting the desired genes were selected. Following optimization of qRT-PCR conditions for each primer set, the actual experiments were performed.

A qRT-PCR reaction mix was prepared for each gene. To summarize, each reaction contained 2 μL of cDNA, 5 pmol of forward and reverse primers, and 5 μL of Bio-Rad’s 2X SYBR Green Supermix. The steps included in the qRT-qPCR procedure were as follows: 10 min of initial denaturation at 95 °C, 30 s of denaturation at 95 °C, 30 s of annealing at 60 °C, and 30 s of extension at 72 °C. This program was run for 40 cycles and qRT-PCR analysis was performed using the Bio-Rad CFX ConnectTM Real-Time System. A melting curve step upon initial amplification of PCR products was included by gradually heating the temperature from 65 °C to 95 °C. ACTB was used as an internal control in our qRT-PCR experiments.

### 2.4. Western Blotting Analysis

Western blotting analysis was used to quantify the levels of caspase-3 (CASP3) and cleaved-CASP3 proteins, using GAPDH as reference. Using a RIPA buffer containing a protease inhibitor cocktail, total protein was extracted from the A549 cell line. After 50 μg of protein was loaded onto polyacrylamide gels, samples were transferred onto a PVDF membrane. Following blocking of the membranes with milk for 1 h at room temperature, rabbit anti-caspase-3 (1:1000; Cat.No sc-98785, Santa Cruz, TX, USA), which was able to recognize both pro- and cleaved-forms of CASP3, and anti-GAPDH (1:700; Santa Cruz, Cat.No sc-25778, TX, USA) primary antibodies were incubated with the membranes overnight at 4 °C. The next day, 0.1% TBST (tris-buffered saline with tween 20) was used to remove excess and unbound antibodies, and then the membranes were incubated with anti-rabbit IgG-HRP secondary antibody (1:5000; Santa Cruz, Cat.No sc-2054, TX, USA). Following the TBST washing steps, protein bands were identified using Chemiluminescent Substrate (Rockford, AZ, USA) under a Bioanalytical Imaging System c280 (Azure Biosystems, Dublin, CA, USA).

### 2.5. CASP3 Activity Assay

CASP3 activity of cells was determined using the CASP3 Colorimetric Assay Kit (BostonChem, Cambridge, MA, USA), according to the supplier’s manual. Wells were designated for a diluted standard, blank, and sample. Seven wells were prepared for the standard and one well for the blank. A total of 100 µL of standard working solution was added to each well. Microplate lids were used to cover the plate. It was incubated for 50 min at 37 °C. Then, the solution in each well was aspirated and each well was washed with 200 µL of 1x washing solution and incubated for 1–2 min. The remaining liquid was completely removed from the wells by placing the plate on absorbent paper. It was then washed three times with washing solution. After the last wash, the remaining wash buffer was aspirated. The plate was inverted and dried with absorbent paper. A total of 100 µL of biotinylated antibody working solution was added to each well and incubated for 50 min at 37 °C. The washing process was repeated five times, followed by aspiration. Afterwards, 90 μL of TMB substrate solution was added to each well and the plate was covered with a new lid. It was incubated in the dark chamber for 20 min at 37 °C. The liquid in the wells turned blue with the addition of the TMB substrate solution. A total of 50 μL of stop reagent was added to each well, and then the liquid in the wells turned yellow. The water droplets and fingerprints at the bottom of the plate and the air bubbles on the surface of the liquid were removed. The measurements were performed at 450 nm via the microplate reader.

### 2.6. Biochemical Analysis of Redox Balance

Total antioxidant status (TAS) and total oxidant status (TOS) of Odo A-treated and control lung cancer cells were determined using human TAS (SanRedBio SRB Technology Campany, Cat.No 201-12-7412, Shanghai, China) and TOS (SanRedBio SRB Technology Campany, Cat.No 201-12-5539, Shanghai, China) ELISA kits. The oxidative stress index (OSI) value was calculated via dividing TOS by TAS levels.

The TAS levels in the control and experimental groups were measured following the manufacturer’s instructions, based on the automated colorimetric method developed by Erel (2004). This procedure quantifies the antioxidative capacity of the analyzed sample against potential reactive oxygen species (ROS) reactions that were sparkled by formed hydroxyl free radicals (OH·). The obtained findings were expressed in µmol Trolox Eq/L as a unit of quantity [20].

The TOS levels in the control and experimental groups were also determined by following the automated colorimetric techniques developed by Erel (2004) [20]. The spectrophotometrically measured color intensity was correlated with the amount of total oxidant molecules found in the sample. Hydrogen peroxide (H_2_O_2_) free radicals were used to calibrate the assay and the findings were expressed in µmol H_2_O_2_ Eq/L as a unit of quantity [21].

The computation of the OSI value was as follows: the ratio of TOS to TAS levels was calculated to determine the OSI value, which was expressed in arbitrary units as a measure of quantity [20].

OSI (arbitrary unit) = TOS (µmol H_2_O_2_ Eq/L)/TAS (µmol Trolox Eq/L).

### 2.7. Protein–Protein Interaction Network Analysis

To better illustrate the functional links among apoptosis-related genes/proteins, specifically involving extrinsic pathway of apoptosis, a protein–protein interaction network of target genes was constructed using the STRING (https://string-db.org/) database. This database utilizes text mining, databases, coexpression, experimentally verified and neighborhood interactions to build an interaction network among multiple proteins. The K-means algorithm was used for clustering of the constructed network to identify functional modules. The K-means algorithm is a widely applied clustering approach for anomaly-based intrusion detection. It attempts to classify a provided set of data into k (a previously defined number) categories [22,23].

### 2.8. Gene Expression Analysis in Lung Adenocarcinoma and Normal Tissues

The Gene Expression Profiling Interactive Analysis (GEPIA) bioinformatics tool (http://gepia2.cancer-pku.cn/) was utilized to analyze differences between gene expression levels of tumor and normal samples [24]. The transcript levels in tumor tissues were retrieved from The Cancer Genome Atlas (TCGA) dataset, while the expression levels in healthy samples were sourced from the Genotype Tissue Expression (GTEx) project. Boxplot diagrams were automatically produced via the GEPIA platform. The cut-off values for *p*-value and log2 fold change were set to 0.05 and 1.5, respectively.

### 2.9. Assessment of Expression and Prognostic Potential of Genes

Kaplan–Meier (KM) estimates of overall survival, disease-specific survival, and progression free intervals for CASP3, CASP7, CASP8 and CASP9 were calculated using the UCSC Xena browser (https://xena.ucsc.edu/) [25]. Data analysis was restricted to primary LUAD tumors from the TCGA dataset, where squamoid and bronchioid subtypes were specifically studied. The gene expression data were split into two groups by median (“high” expression ≥ median and “low” expression < median). *p*-values less than 0.05 were considered as statistically significant.

### 2.10. Statistical Analysis

The 2^−ΔΔCt^ technique was used to calculate relative fold changes in gene expression levels of samples for qRT-PCR study. The relative levels of pro- and cleaved-CASP3 were normalized to GAPDH in Western blotting experiments. The statistical analysis of qRT-PCR results, Western blotting, ELISA data, TAS and TOS levels, and OSI values were performed via the GraphPad Prism statistics tool (Version 8.0.2; GraphPad software Inc., San Diego, CA, USA). The normality of sample distribution was tested using the Shapiro–Wilk test. The parametric variables were analyzed through student’s *t*-test while nonparametric variables were compared using the Mann–Whitney U test. Pearson’s correlation analysis comparing all investigated apoptotic genes with each other was performed using GraphPad Prism (Version 10; GraphPad software Inc., San Diego, CA, USA). A Pearson’s correlation coefficient (r) value between 0 and 1 indicates a positive correlation, while r between −1 and 0 reflects negative correlation between two compared genes. Correlogram analysis of our results was also performed. In every analysis, *p*-values < 0.05 were deemed as statistically significant.

## 3. Results

### 3.1. Odo A Inhibited the Proliferation of Lung Cancer Cells

**Figure 1:**
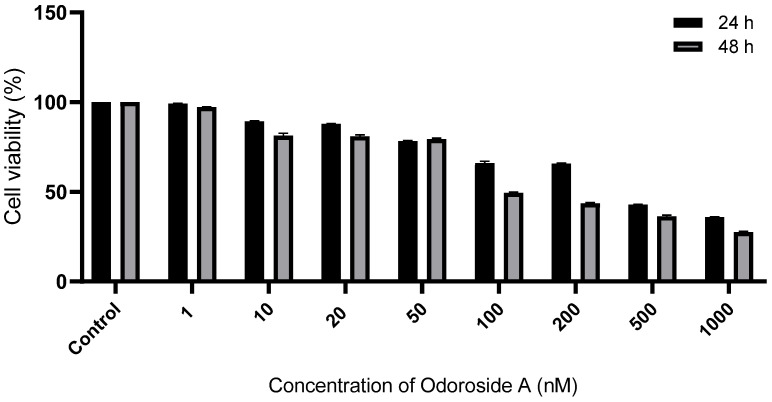
Cytotoxic effect of Odo A on A549 lung cancer cells at 24 and 48 h. Growth inhibition on A549 cells after 24 and 48 h treatment by different concentrations of Odo A ranging from 0–1000 nm was evaluated via CCK-8 assay. IC_50_ value of Odo A for A549 cells was calculated as 183.5 nM at 48 h via CompuSyn version 1.0 software.

A549 cells were treated with a concentration range of Odo A (0–1000 nM) for 24 and 48 h. It was found that, as the dose increased, the viability of lung cancer cells decreased (Figure 1). Using CompuSyn version 1.0 software, the half maximum inhibitory concentration (IC_50_) of Odo A for A549 cells was determined as 183.5 nM at 48 h. Thus, A549 cells were treated with a concentration of Odo A corresponding to its calculated IC_50_ value in the following experiments.

### 3.2. Treatment of Lung Cancer Cells with Odo A Activated the Extrinsic Apoptotic Pathway but Not the Intrinsic Apoptotic Pathway

**Figure 2:**
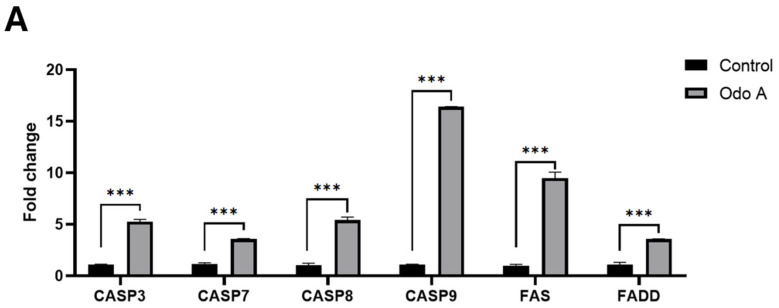

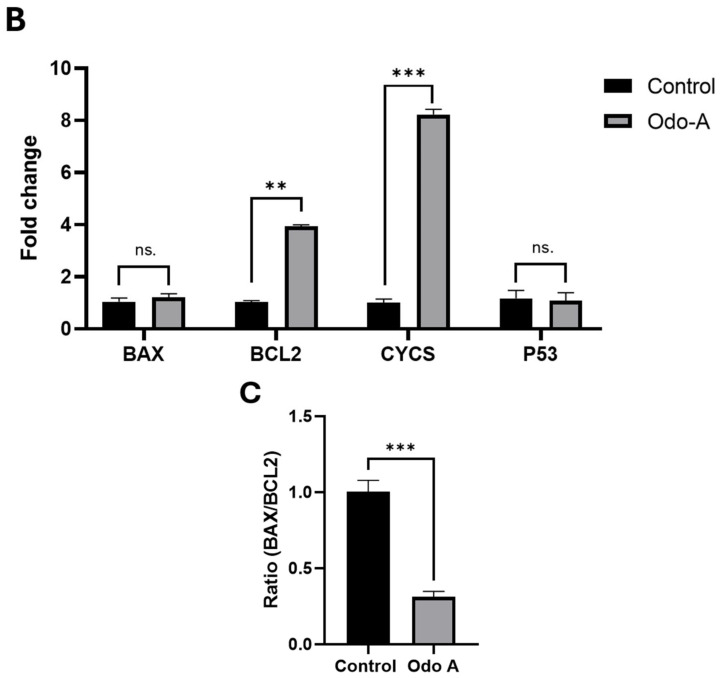
Expression changes of extrinsic apoptotic pathway-related genes (**A**), intrinsic apoptotic pathway-related genes (**B**) and the ratio of *BAX* to *BCL2* (**C**). Effect of Odo A on *CASP3*, *CASP7*, *CASP8*, *CASP9*, *FAS*, *FADD*, *BAX*, *BCL2*, *CYCS*, and *P53* mRNA levels in A549 lung cancer cells after treatment with IC_50_ for 48 h. The relative quantification of the target genes was performed using the 2^−ΔΔCt^ method. Beta-actin was used as a housekeeping gene in qRT-PCR experiments. Bar graphs representing the fold changes in control and treatment groups were drawn with GraphPad Prism (Version 8.0.2, GraphPad software Inc., San Diego, CA, USA). Obtained data were analyzed using student’s *t*-test. *p*-values < 0.05 were considered as statistically significant. *p*-values between 0.033 and 0.02 are designated with (**). *p*-values < 0.001 are designated with (***). *p*-values > 0.05 are considered as statistically not significant (ns.). Each group had three biological replicates.

Based on our qRT-PCR results of Odo A-treated A549 cells, statistically significant changes were obtained in the expression levels of *CASP3*, *CASP7*, *CASP8*, *CASP9*, *FAS* and *FADD* genes, especially those involved in the extrinsic apoptotic pathway, as compared to the control cells (Figure 2A, *p* < 0.05). Therefore, Odo A treatment has been shown to trigger apoptosis in lung cancer cells via the extrinsic pathway.

According to *BAX* and *BCL2* gene expression analysis, which are related to the other triggering mechanism of the apoptotic pathway (the intrinsic pathway), the change in the expression level of the *BAX* gene was not significant (Figure 2B, *p* > 0.05), while a remarkable increase in the *BCL2* gene was detected (Figure 2B, *p* < 0.05) in Odo A-treated cells as compared to the control group. Moreover, the BAX/BCL2 ratio, that gives information about the cell death switch, was found to significantly decrease in Odo A-treated cells (Figure 2C, *p* < 0.05). Our results clearly indicated that the treatment of A549 cells with Odo A did not induce intrinsic apoptosis.

To further elucidate the molecular mechanisms behind cell viability and apoptosis induction in response to Odo A treatment, we examined the gene expression levels of key players of the PI3K/AKT signaling pathway. The mRNA transcripts of *AKT1* and *PTEN* were upregulated (*p* < 0.05; Supplementary Data S1), while *GSK3ß*, *CCND1* and *P21* levels were downregulated (*p* < 0.05; Supplementary Data S1) in Odo A-treated lung cancer cells compared to the control group. As expected, upregulated AKT further inhibited the downstream *GSK3ß*, *CCND1* and *P21* levels, which were all confirmed by our data. Thus, the PI3K/AKT signaling pathway played a crucial role in the regulation of cell survival and apoptosis induction following Odo A treatment in lung cancer cells. Surprisingly, an inhibitory effect of AKT1 on PTEN was not shown, suggesting the involvement of converging pathways in the activation of apoptosis. The primer sequences of these genes are provided in Supplementary Table S1.

### 3.3. Odo A Treatment Led to Increased CASP3 Expression and Activity in Lung Cancer Cells

**Figure 3:**
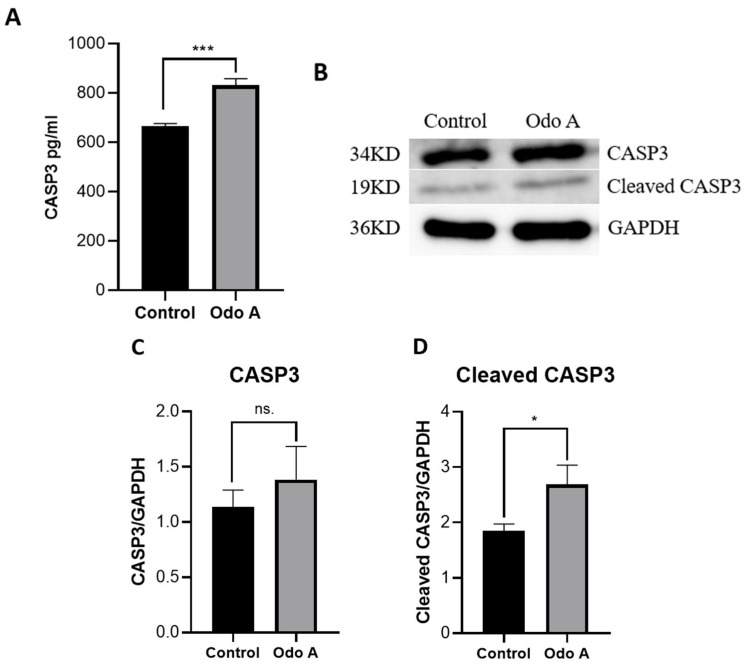
Detection of CASP3 activity and protein levels in Odo A-treated and control A549 cells using the Western blotting method. (**A**) CASP3 activity was measured in the two groups using ELISA. (**B**) Western blotting images of pro- and cleaved-CASP3 and GAPDH are shown. Relative densitometric bar graphs of pro-CASP3 (**C**) and cleaved-CASP3 (**D**) were calculated with GraphPad Prism (Version 8.0.2, GraphPad software Inc., San Diego, CA, USA). Data are expressed as mean ± standard deviation (SD) in each group and analyzed using student’s *t*-test. *p*-values < 0.05 are considered as statistically significant. *p*-values between 0.05 and 0.033 are designated with (*), *p*-values between 0.033 and 0.02 are designated with (**), and *p*-values < 0.001 are designated with (***). *p*-values > 0.05 are considered as statistically not significant (ns.). Each group had three biological replicates.

Our results showed that the mRNA transcript level of *CASP3* increased significantly in the Odo A treatment group compared to control cells (Figure 2A; *p* < 0.05). This result led us to check whether it was reflected at the levels of protein and activity. Initially, CASP3 activity was tested in the experimental and control groups. Our ELISA results indicated that the activity of CASP3 was significantly stimulated by Odo A treatment in A549 cells (Figure 3A; *p* < 0.05). Furthermore, our Western blotting analysis also confirmed the previous findings by showing significant elevation in the cleaved CASP3 (active CASP3) levels in the treatment groups (Figure 3B,D; *p* < 0.05). There was also an increasing trend in pro-CASP3 levels. However, this change in Odo A-treated cells was not significantly different from the control cells (Figure 3B,C; *p* > 0.05). Overall, the treatment of A549 cells with Odo A increased the protein level and activity of CASP3 (Figure 3). Therefore, we can confidently claim that changes in *CASP3* transcript levels were translated to the protein.

### 3.4. The Effect of Odo A Treatment on Redox Balance of Lung Cancer Cells

**Figure 4:**
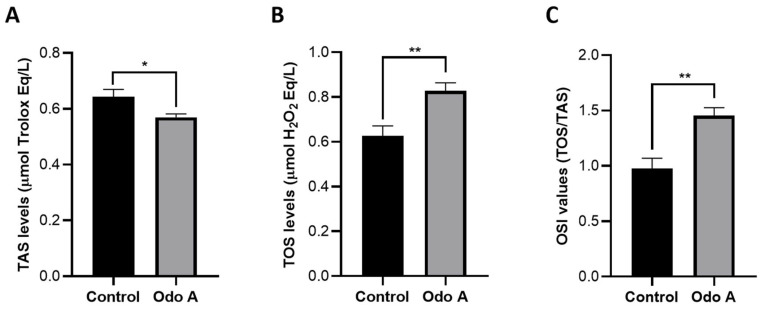
Amounts of TAS and TOS in A549 cells treated with Odo A (**A**,**B**), and ratio of TAS and TOS to each other (**C**). Levels of TAS (**A**) and TOS (**B**) and the calculated TOS/TAS ratio and OSI values (**C**), in the control and Odo A-treated A549 lung cancer cell lines were calculated using GraphPad Prism (Version 8.0.2, GraphPad software Inc., San Diego, CA, USA). Parametric data were analyzed using student’s *t*-test. Results are shown as means ± SEM and *p*-values < 0.05 are considered as statistically significant. *p*-values between 0.05 and 0.033 are designated with (*), and between 0.033 and 0.02 are designated with (**). Each group had three biological replicates.

Odo A-treated A549 cells had significantly lower TAS levels than control cells, showing decreased antioxidant capacity in the experimental group (Figure 4A, Supplementary Table S2; *p* < 0.05). TOS levels represent the prooxidant status of cells. Based on our data, TOS levels significantly increased in Odo A-treated lung cancer cells as compared to control cells, indicating elevated oxidative stress caused by our treatment (Figure 4B, Supplementary Table S2; *p* < 0.05). Moreover, the calculated OSI values were also significantly higher in treatment group than control samples (Figure 4C, Supplementary Table S2; *p* < 0.05). Low TAS levels, high TOS levels and high OSI values showed overall oxidative stress in Odo A-treated lung cancer cells. The disruption of redox homeostasis in favor of prooxidant production might be linked to the induction of apoptosis due to our treatment (Figure 2 and Figure 3).

### 3.5. Apoptotic Pathway Genes Showed Strong Correlations and Interactions

**Figure 5:**
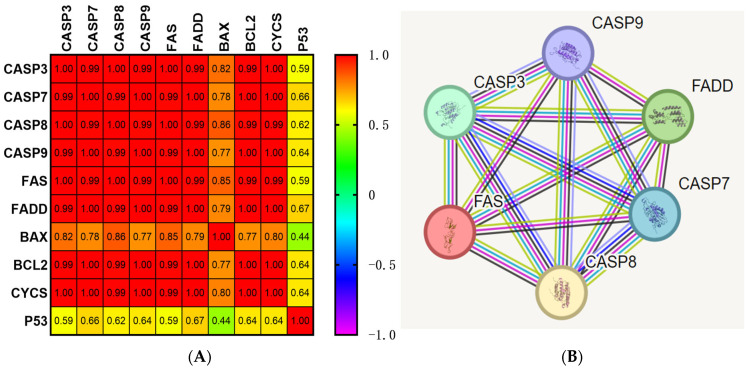
(**A**) Correlogram of qPCR-screened apoptosis-related genes showing positive and high correlations with each other. Pearson correlation analysis was performed using fold change values of target genes in both control and Odo A treatment groups through GraphPad Prism (Version 10; GraphPad software Inc., San Diego, CA, USA). Numbers in each square represent correlation coefficients (r) for the corresponding two genes. Color key for the correlogram coefficients is indicated at the right side of the plot. All correlations among genes except P53 are significant, with *p*-values < 0.05. (**B**) The Protein–protein interaction network of qPCR-screened target genes involved in extrinsic pathway of apoptosis, namely *CASP3*, *CASP7*, *CASP8*, *CASP9*, *FAS*, and *FADD*, was drawn using STRING v12. Each network node represents a gene. Black lines denote confirmed coexpression, signifying a functional link among proteins, while green lines show interactions based on text mining. Dark blue lines predict interaction based on gene co-occurrence, while light blue lines indicate protein homology. Cyan and purple lines show known interactions from curated databases and experimentally-determined results, respectively. The calculated interaction score was set to greater than 0.4.

The correlation analysis of all investigated genes demonstrated positive and significantly high correlations among them (Figure 5A, Supplementary Data S2; *p* < 0.05). Although P53 had a positive and relatively high correlation with other genes, this was not significant (Figure 5A, Supplementary Data S2; *p* > 0.05). Overall, the correlogram clearly displayed strong functional relationship between apoptotic genes. The functional impacts of target genes were also presented in system levels analysis by constructing a protein–protein interaction (PPI) network. The constructed network consisted of 6 nodes (extrinsic apoptotic pathway-related genes) and 15 edges, where the strength of interaction score was adjusted to greater than 0.4 (the PPI enrichment *p*-value was 1.86 × 10^−12^). The STRING PPI network analysis revealed significant functional links and close associations among the qPCR-screened genes (Figure 5). The black colored line between all the investigated extrinsic pathway genes confirmed the functional links among them. Moreover, the interconnected interactome of these genes also proved the obtained high correlations and associations in the previously presented heatmap. The functional relationships among the genes explained their similar upregulation patterns in response to Odo A treatment to activate the extrinsic pathway of apoptosis (Figure 2A and Figure 5B). 

### 3.6. No Differential Expression Profiles of Apoptotic Genes Were Observed Between Lung Adenocarcinoma and Healthy Tissues

**Figure 6:**
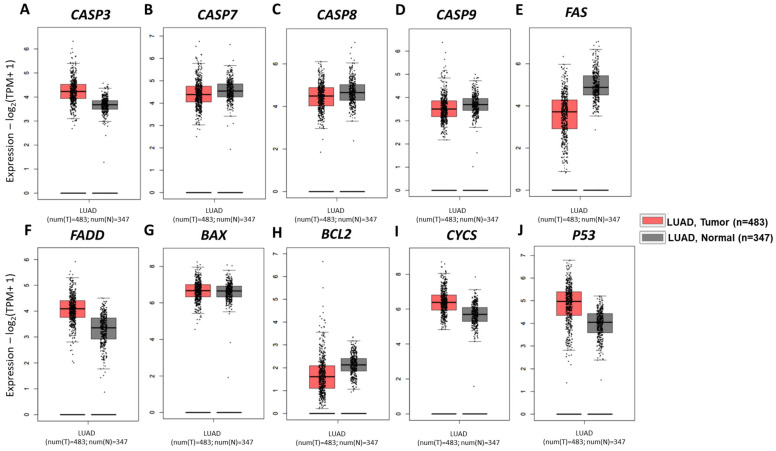
Expression analysis of apoptosis-related genes performed via GEPIA2 in LUAD samples (red, T = 483) and normal tissue (grey, N = 387) from TCGA and GTEx datasets, respectively. The (log2 (TPM+1)) transformed gene expression data were used in graphical representations for (**A**) *CASP3*, (**B**) *CASP7*, (**C**) *CASP8*, (**D**) *CASP9*, (**E**) FAS, (**F**) FADD, (**G**) BAX, (**H**) BCL2, (**I**) *CYCS*, and (**J**) *P53*. *p*-values < 0.05 are considered statistically significant.

Differences in the transcript levels of genes are commonly encountered between normal and tumor tissues. The expression amounts of oncogenic genes are mostly increased, while tumor-suppressor genes show a declining pattern in many cancer types [26]. This fact encouraged us to examine the differential expression profiles of target genes between LUAD and normal tissues. The transcript levels of extrinsic apoptotic pathway markers, namely *CASP3*, *CASP7*, *CASP8*, *CASP9*, *FAS*, and *FADD*, did not show any significant differences in LUAD samples as compared to healthy lung tissues (Figure 6A–F, *p*-values > 0.05). Moreover, there were also no remarkable differences in the expression levels of *BAX*, *BCL2*, *CYCS*, and *p53* genes between cancerous and normal lung tissues (Figure 6G–J, *p*-values > 0.05). All these results emphasized one important point, that neither the extrinsic nor intrinsic apoptotic pathways can be activated spontaneously in lung cancer. Thus, the activation of the extrinsic, not the intrinsic apoptotic pathway, in A549 lung cancer cell was triggered by the Odo A treatment in our study (Figure 2).

### 3.7. Caspases Showed No Prognostic Value in Lung Cancer, Independent of Treatment

**Figure 7:**
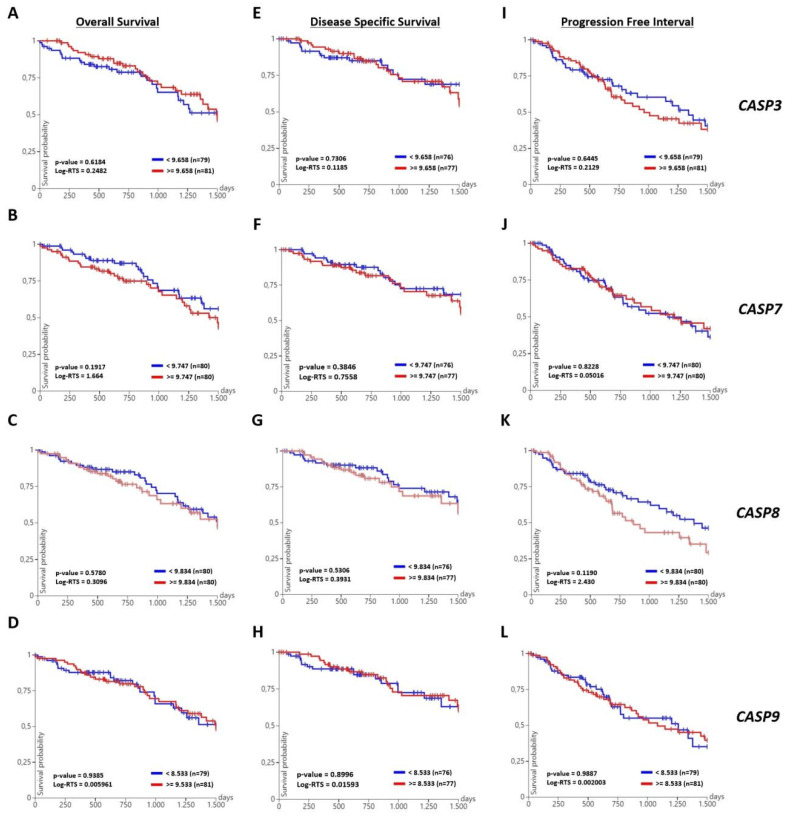
The prognostic value of *CASP3*, *CASP7*, *CASP8*, and *CASP9* were investigated in LUAD. KM plots were sketched using the UCSC Xena database from the TCGA LUAD dataset. KM estimates of overall survival for (**A**) *CASP3*, (**B**) *CASP7*, (**C**) *CASP8*, and (**D**) *CASP9* are shown. KM estimates of disease-specific survival for (**E**) CASP3, (**F**) CASP7, (**G**) CASP8, and (**H**) *CASP9* are indicated. Kaplan–Meier estimates of progression free interval for (**I**) *CASP3*, (**J**) *CASP7*, (**K**) *CASP8*, and (**L**) *CASP9* are presented. The log-rank test (RTS) was applied to compute *p*-values. *p*-values < 0.05 are considered statistically significant.

To investigate the prognostic value of CASP family members, KM analysis was performed for *CASP3*, *CASP7*, *CASP8*, and *CASP9* genes in primary LUAD tumor samples, with different survival outcomes. However, our results did not reveal a significant relationship between high/low expression levels of these genes and overall survival (OS) (Figure 7A–D), disease-specific survival (DSS) (Figure 7E–H) or progression free interval (PFI) (Figure 7I–L) in LUAD patients. Therefore, it might be concluded that caspases were not associated with the prognosis of lung cancer, including LUAD.

## 4. Discussion

All of the typical tissues of multicellular organisms must have a balance between cell proliferation and cell death. Apoptosis is the term used to describe this process of natural cell death, which is essential for cell growth and proliferation and involves the cascades of coordinated processes. The last stage in each route is the activation of caspases. Because apoptosis is the primary mechanism of cell death, it is widely studied in anti-cancer therapies. For this reason, the effects of agents with anti-tumorigenic activity on apoptotic pathways should be investigated first. The main purpose of our study was to reveal how Odo A, which is obtained from the lily plant, induces apoptosis in lung cancer cells. Moreover, we were also aimed to comprehensively support our in vitro finding by utilizing in silico-based approaches.

Potent natural substances like cardiac glycosides are frequently used in the treatment of cardiovascular diseases such as arrhythmia, myocardial ischemia, and congestive heart failure. Notably, over the past few years, cardiac glycosides have been discovered to possess potential anti-cancer properties. Furthermore, cardiac glycosides have the ability to cause cancer cells to die, via controlling proteins linked to apoptosis [27]. According to earlier research, Odo A reduced cell activity in human cancer cell lines, including cervical, colon, and stomach cancers [10]. In our study, we showed that Odo A activated apoptosis in lung cancer cells. Based on data presented in previous in vitro studies, cardiac glycosides are safe for normal cells at nanomolar doses and could even prevent their apoptosis or promote cell growth. However, these medications were found to inhibit cell growth and cause cell death in cancer cells [28,29,30]. Mono glycosidic cardenolides, namely oleandrin and Odo A, that were isolated from *Nerium oleander*, have been demonstrated to possess strong anti-cancer properties [6,11,12]. The therapeutic effects of Odo A on cancer types are less well understood than those of oleandrin. Here, we initially applied a CCK-8 assay to examine Odo A’s anti-tumorigenic activities. Potent inhibitory effects of Odo A were observed on the proliferation of lung cancer cells.

When apoptosis is induced externally, the death effector domains (DED) of FAS and FADD in the cell membrane trigger the activation of a series of caspase proteins. The death-inducing signaling complex (DISC) during apoptosis is formed by the adaptor protein FADD, which connects procaspases 8 and 10 to the members of the tumor necrosis factor receptor superfamily, including the FAS-receptor. A study showed that the bridge formed by the FAS and FADD proteins is effective in the activation of the CASP8 protein [30]. Numerous illnesses, including cancer, are strongly correlated with disruption in the function of the FADD protein [31,32,33]. A number of solid malignancies, such as gliomas, NSCLC, and hepatocellular carcinoma (HCC), have been shown to express FADD aberrantly [34,35]. Due to its effects on various aspects of cancer cell behavior, including proliferation, apoptosis, the cell cycle, autophagy, inflammation, and drug resistance, dysregulation in FADD activity leads to the progression of cancer [36,37]. In our study, it was found that FAS, FADD, and CASP8 increased significantly, suggesting that Odo A stimulated the extrinsic apoptotic pathway.

Another component that is intimately linked to apoptosis is proteins belonging to the caspase family. CASP8 and CASP9 are the initiator caspases in the extrinsic and intrinsic apoptotic pathways, respectively, and CASP3 is an executioner caspase in both cases [38]. Our qRT-PCR results indicated that Odo A treatment led to upregulation of *CASP3*, *CASP7*, *CASP8*, and *CASP9* expression, which suggests that the extrinsic route was responsible for blocking the development of lung cancer cells (Figure 2A). Moreover, elevated activity and protein levels of activated CASP3 (cleaved-CASP3) confirmed our qPCR findings, as seen in Figure 3.

The release of CYCS from the mitochondrial intermembrane space into the cytoplasm, as well as mitochondrial depolarization, are linked to the intrinsic pathway, which is activated in response to DNA damage. A complex known as the apoptosome is then formed by CYCS, apoptotic protease-activating factor 1 (APAF1), and pro-CASP9. Within this complex, the activation of CASP9 facilitates the cleavage, and subsequently activation of CASP3, CASP6 and CASP7 [39]. However, one of the first studies on this topic connects the intrinsic and extrinsic apoptotic pathways, supporting the notion that there are converging pathways rather than separate ones [40,41]. Later studies showed that the intrinsic and extrinsic pathways are interconnected. In our study, the p53 gene expression increased, although the changes were not significant. Bouvard et al. (2000) reported that p53-associated Fas mRNA increase has been shown in the lung, spleen, thymus, and kidney [42]. Overexpressed p53 might promote trafficking of the FAS receptor from the Golgi, resulting in an increased FAS mRNA transcript level at the cell surface [43]. This might make cells more susceptible to FAS-induced apoptosis quickly, through p53, before the transcription-dependent effect takes place. Although p53 does not appear to be directly related to the extrinsic apoptotic pathway, it is possible that it somehow overlaps with the intrinsic pathway. It has been demonstrated that Odo A can cause colorectal cancer cells to undergo mitochondrial apoptosis via the p53 pathway [44]. However, this was not corrected for lung cancer, as indicated in Figure 2B.

To further clarify the biochemical mechanisms behind cell survival and apoptosis induction in response to Odo A administration in A549 cells, we also examined the gene expression levels of critical components of the PI3K/AKT signaling pathway. According to our results, *AKT1* expression was shown to be significantly increased in Odo A-treated lung cancer cells (Supplementary Data S1; *p* < 0.05). AKT is the major player in the PI3K/AKT/mTOR signaling pathway, has been linked to a number of diseases induced by abnormal cell proliferation, and has been demonstrated to play significant roles in controlling cell viability. Nevertheless, the specific molecular processes AKT1 uses to control apoptosis to encourage cell death are still mostly unknown [45,46]. It was observed that the increase in the expression of *AKT1* was accompanied by a significant decrease in *GSK3ß*, *p21*, and *CCND1* (Supplementary Data S1; *p* < 0.05). This showed a suppressive effect of *AKT1* upregulation on cell proliferation. When the expression of *PTEN*, a tumor suppressor gene, was analyzed, it was found to be significantly increased (Supplementary Data S1; *p* < 0.05). It could be suggested that Odo A treatment increased *PTEN* expression in lung cancer cells, thus stimulating apoptotic pathways, independent of AKT1 signaling [47].

Under certain conditions, ROS, which are very reactive chemicals that are typically formed as byproducts of oxygen metabolism in the human body, regulate the integrity of DNA repairment processes. They are also thought to have a dual role in the development and prevention of cancer. A high quantity of ROSs stimulates cell death by activating apoptosis and/or autophagy, even while a moderate level of free radicals preserves the vital mechanisms of cancer cell survival [48,49]. The biochemical analysis of oxidative stress provided comparable results with those of the aforementioned studies. Based on our data, disturbance in redox balance in the favor of prooxidant production might be related to the activation of apoptosis in Odo A-treated lung cancers cells (Figure 4, Supplementary Table S2).

Our bioinformatics analysis also provides information supporting our experimentally-proven findings. STRING analysis displayed direct interactions within close neighborhoods among all studied genes (Figure 5B, Supplementary Figure S1). These data revealed functional and regulatory relationships among genes, not only in apoptotic pathways but also in other converging pathways. For example, the interactome clearly explained that the binding of pro-CASP8 to FADD eventually led to activation of CASP8, while the interaction of CYCS and CASP9 together with APAF1 was involved in the formation of the apoptosome (Figure 5B, Supplementary Data S2) [39,50]. We also looked at whether the investigated genes were differentially expressed between LUAD and healthy tissues. Based on our GEPIA analysis, neither extrinsic nor intrinsic apoptotic pathway-related genes were expressed significantly differently in LUAD patients compared to normal individuals (Figure 6). Spontaneous apoptosis often occurs at too low a level and, ultimately, causes malignant transformation of affected cancerous cells, so that primary tumors start to metastasize to other parts of the body [51,52]. Therefore, insufficient apoptosis in LUAD tissues might be stimulated through Odo A treatment, as shown in Figure 2. KM graphs of CASP3, CASP7, CASP8, and CASP9 did not show a significant association between high/low expression profiles of referred genes and OS, DSS, and PFI. However, targeting caspases in cancer therapies e.g., in prostate cancer, has led to the highest survival rates of patients [53,54]. Although our results revealed no prognostic value of caspases in LUAD, we have successfully indicated their activator role in apoptosis, particularly in extrinsic apoptosis, which enabled us to propose them as potential therapeutic targets of Odo A in lung cancer cells.

Previous literature indicates anti-tumor effect of Odo A and its derivative, oleandrigenin-3-O-β-D-diginoside, on both HL60 and K562 human leukemia cell lines via activation of the ROS/p53 pathway [12]. In the current work, we described a different mechanism of action for the induction of apoptosis by activation of the extrinsic pathway in the Odo A-treated A549 lung cancer cell line. This proved that the same chemical compound can still yield a significant anti-cancer effect by targeting different signaling molecules in various types of cancers. Despite its promising therapeutic potential, Odo A is not currently included in established pharmacogenomic databases, such as the Genomics of Drug Sensitivity (GDSC). Therefore, we believe in that our research will contribute to the future construction of Odo A-treated A549 datasets in the GDSC library. A key limitation of this study was the use of A549 cells as an in vitro model for lung adenocarcinoma. The treatment of A549 cells with Odo A might not entirely reflect the physiological responses of normal lung epithelial cells. Therefore, our results may possess limited translational significance for healthy lung tissue. However, we intended to test the apoptotic and prooxidative effects of Odo A to mainly reveal its therapeutic potential on lung cancer cells. Nonetheless, it is strongly suggested that further validation with human lung epithelial cells is performed, in order to strengthen the conclusions of this study.

## 5. Conclusions

The optimal dosage of Odo A for lung cancer cells was determined as 183.5 nM, which was used in our experiments to test the hypothesis of the current study. Based on qRT-PCR analysis of Odo A-treated lung cancer cells, we found significant increases in the mRNA transcript levels of apoptotic genes, specifically those involved in the extrinsic apoptotic pathway. However, our data did not show the activation of the intrinsic apoptotic pathway. Therefore, Odo A treatment of lung cancer cells induced extrinsic, but not intrinsic apoptosis. Moreover, we have also successfully verified obtained gene expression data at the protein and activity levels. Furthermore, we explained excessive production of prooxidants as a mechanistic activator of apoptosis in Odo A-treated lung cancer cells. Additional bioinformatics analysis supported our in vitro results by showing strong correlations and interactions among genes. Further differential gene expression and KM analyses of target genes neither detected significant changes between LUAD and healthy tissues nor was able to verify them as good prognostic biomarkers. Last but not least, the presented results revealed the therapeutic effect of Odo A on lung cancer cells, enabling the translation of this finding into its use in clinics to cure patients suffering from LUAD, NSCLC, or SCLC. 

## Figures and Tables

**Table 1:** Primers used in qRT-PCR analysis.

Genes	Primer Sequences	PCR Product Length (bp)
BAX	F: GGAGCTGCAGAGGATGATTG R: GGCCTTGAGCACCAGTTT	151
BCL2	F: GTGGATGACTGAGTACCTGAAC R: GAGACAGCCAGGAGAAATCAA	125
CASP3	F: GAGCCATGGTGAAGAAGGAATA R: TCAATGCCACAGTCCAGTTC	162
CASP7	F: CGAAACGGAACAGACAAAGATG R: TTAAGAGGATGCAGGCGAAG	169
CASP8	F: GCCCAAACTTCACAGCATTAG R: GTGGTCCATGAGTTGGTAGATT	160
CASP9	F: CGACCTGACTGCCAAGAAA R: CATCCATCTGTGCCGTAGAC	153
CYCS	F: GGAGAGGATACACTGATGGAGTA R: GTCTGCCCTTTCTTCCTTCTT	102
P53	F: GAGATGTTCCGAGAGCTGAATG R: TTTATGGCGGAGGTAGACT	129
FAS	F: GTGATGAAGGACATGGCTTAGA R: GTGTGCATTCCTTGATGATTCC	156
FADD	F: TGACCGAGCTCAAGTTCCTATG R: CCAGGTCGTTCTGCTCCAG	108
ACTB	F: TGGCTGGGGTGTTGAAGGTCT R: AGCACGGCATCGTCACCAACT	179

## Data Availability

Data used/analyzed in this current study are available from the corresponding author upon request. Correspondence and requests for materials and data should be addressed to F.S.C.

## Supplementary Materials

The following supportive information data can be downloaded at: https://www.mdpi.com/article/10.3390/life15030445/s1.
