# Supplementary material for: Evaluation of the Apoptotic, Prooxidative and Therapeutic Effects of Odoroside A on Lung Cancer: An In Vitro Study Extended with In Silico Analyses of Human Lung Cancer Datasets
Source: Life (Basel). 2025 Mar 12;15(3):445. doi: 10.3390/life15030445 (PMC11944172; doi:10.3390/life15030445)
Supplement: Supplementary file 1 [file life-15-00445-s001.zip › life-3446715-supplementary.pdf]

SUPPLEMENTARY

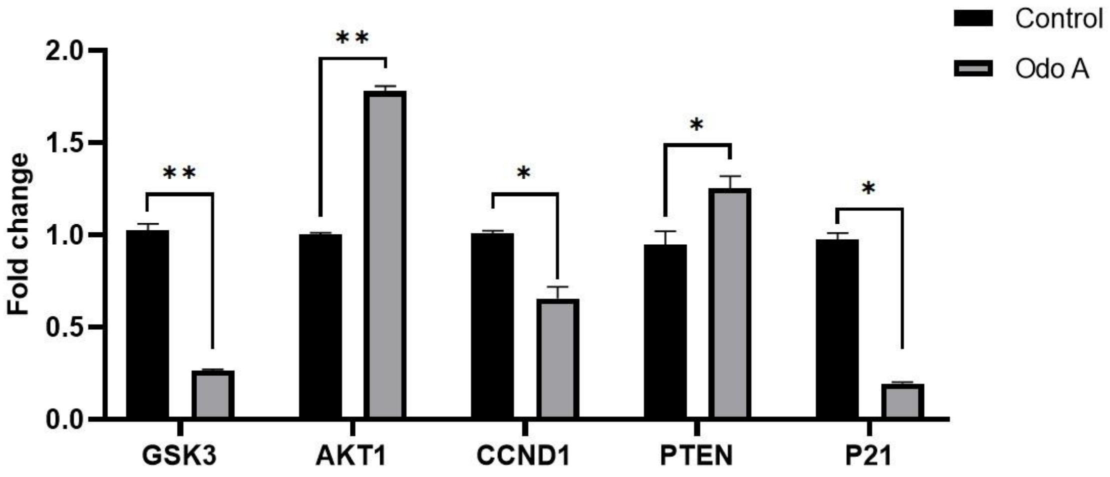

Supplementary Data S1. Expression changes of cell survival pathway-related genes. Effect of Odo A on *GSK3, AKT1, CCND1, PTEN* and *P21* mRNA levels in A549 lung cancer cells after treatment with IC50 for 48 hours. The relative quantification of the target genes was performed using 2^(-ΔΔCt) method. Beta-actin was used as a housekeeping gene in qRT-PCR experiments. Bar graphs representing the fold changes in control and treatment groups were drawn with GraphPad Prism (Version 8.0.2, GraphPad software Inc., San Diego California, USA). Obtained data was analyzed using student's t-test. P-values<0.05 were considered as statistically significant. p-values less than 0.001 were designated with (***). p-values>0.05 were reported as statistically not significant (ns.). Each group had three biological replicates.

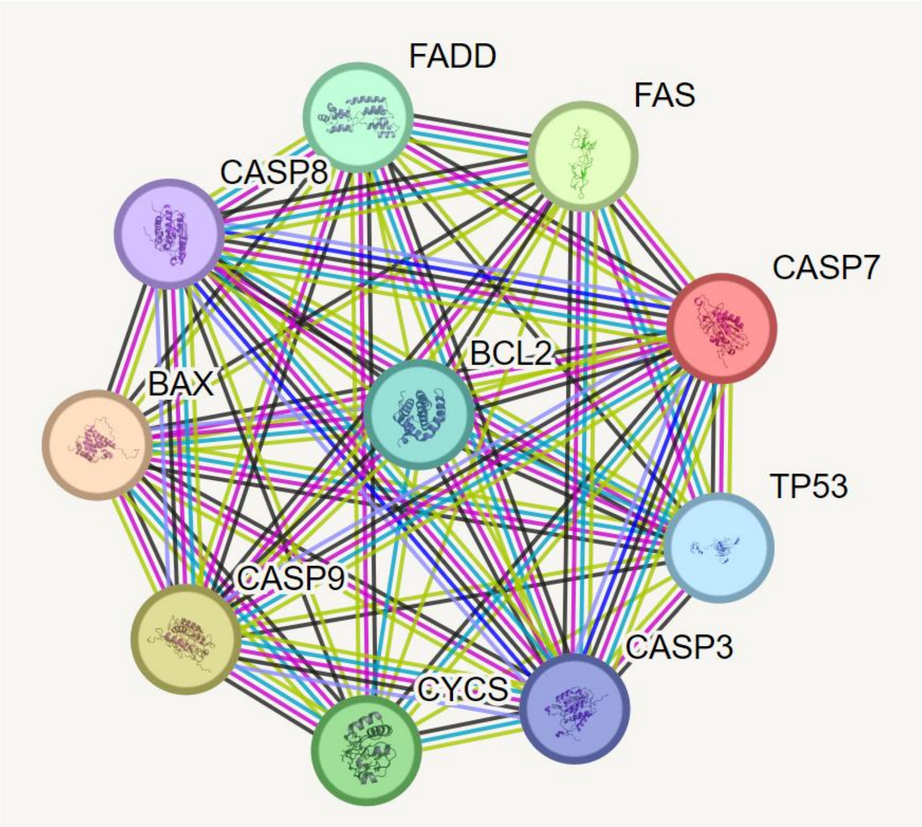

Supplementary Data S2. Protein-protein interaction network of qPCR-screened apoptosis related genes was drawn using STRING v12. The black lines denote confirmed co-expression thereby functional link among proteins while green-colored lines show the interactions based on textmining. Dark-blue lines predict interaction based on gene co-occurance whereas light-blue colored lines indicate protein homology. Moreover, cyan and purple-colored lines show known interactions from curated databases and experimentally-determined results, respectively.

Supplementary Table S1. Primers used in qRT-PCR analysis for major revision.

	Primer sequences	PCR product length (bp)
GSK3B	F: CTAACACCACTGGAAGCTTGT<br>R: TCTTGAGTGGTGAAGTTGAAGAG	113
AKT1	F: CAAGGACGGGCACATTAAGA<br>R: CATTGTCCTCCAGCACCTC	125
PTEN	F: CGTTACCTGTGTGTGGTGATA<br>R: CTCTGGTCCTGGTATGAAGAATG	116
P21 (CDKNA1)	F: TGGACCTGTCACTGTCTTGTA<br>R: AGAAATCTGTCATGCTGGTCTG	120
Cycline D1 (CCND1)	F: GTTCGTGGCCTCTAAGATGAA<br>R: AGGTTCCACTTGAGCTTGTT	135

Supplementary Table S2. Oxidant and antioxidant parameters in Odo A-treated and control lung cancer cells were provided. TAS, total antioxidant status; TOS, total oxidant status; OSI, oxidative stress index.

	**Control group**	**Odo A-treated group**	**p-values**
**TAS (µmol Trolox Eq/L)**	0.6437±0.0151	0.5700±0.0067	0.03 (*)
**TOS (µmol H_2O_2 Eq/L)**	0.6263±0.0258	0.8280±0.0208	<0.01 (**)
**OSI (arbitrary unit)**	0.9748±0.0539	1.4531±0.0414	<0.01 (**)

The results were indicated as ± SEM and p-values < 0.05 were considered as statistically significant. p-values between 0.05 and 0.033 were designated with (*), between 0.033 and 0.02 were designated with (**).

Supplementary Table S3. Pearson correlation coefficient (r) values for qPCR data analysis of target genes are provided. Fold changes of target genes were compared with each other in both control and OdoA treatment groups.

	**CASP3**	**CASP7**	**CASP8**	**CASP9**	**FAS**	**FADD**	**BAX**	**BCL2**	**CYCS**	**P53**
**CASP3**	1	0,994	0,995	0,995	0,999	0,993	0,825	0,995	0,997	0,587
**CASP7**	0,994	1	0,990	0,999	0,990	1,000	0,784	0,999	0,999	0,663
**CASP8**	0,995	0,990	1	0,987	0,998	0,990	0,865	0,987	0,992	0,623
**CASP9**	0,995	0,999	0,987	1	0,990	0,998	0,771	1,000	0,999	0,635
**FAS**	0,999	0,990	0,998	0,990	1	0,990	0,848	0,990	0,994	0,586
**FADD**	0,993	1,000	0,990	0,998	0,990	1	0,788	0,998	0,999	0,672
**BAX**	0,825	0,784	0,865	0,771	0,848	0,788	1	0,772	0,796	0,440
**BCL2**	0,995	0,999	0,987	1,000	0,990	0,998	0,772	1	0,999	0,636
**CYCS**	0,997	0,999	0,992	0,999	0,994	0,999	0,796	0,999	1	0,636
**P53**	0,587	0,663	0,623	0,635	0,586	0,672	0,440	0,636	0,636	1

Supplementary Table S4. Pearson correlation analysis corresponding p-values from the comparison of observed correlation between target genes are provided. Fold changes of target gene were compared with each other in both control and OdoA treatment groups.

	CASP3	CASP7	CASP8	CASP9	FAS	FADD	BAX	BCL2	CCNL2	P53
**CASP3**		5,91E-05	3,08E-05	4,25E-05	1,31E-06	7,31E-05	0,0434	4,25E-05	1,16E-05	0,2204
**CASP7**	5,91E-05		0,0002	1,56E-06	0,0001	1,44E-08	0,0647	1,39E-06	8,26E-07	0,1509
**CASP8**	3,08E-05	0,0002		0,0003	5,90E-06	0,0001	0,0263	0,0003	9,00E-05	0,1863
**CASP9**	4,25E-05	1,56E-06	0,0003		0,0001	4,59E-06	0,0725	9,92E-13	9,40E-07	0,1751
**FAS**	1,31E-06	0,0001	5,90E-06	0,0001		0,0001	0,0328	0,0001	4,69E-05	0,2221
**FADD**	7,31E-05	1,44E-08	0,0001	4,59E-06	0,0001		0,0626	4,21E-06	1,95E-06	0,1440
**BAX**	4,34E-02	0,0647	0,0263	0,0725	0,0328	0,0626		0,0723	0,0584	0,3831
**BCL2**	4,25E-05	1,39E-06	0,0003	9,92E-13	0,0001	4,21E-06	0,0723		8,71E-07	0,1744
**CYCS**	1,16E-05	8,26E-07	9,00E-05	9,40E-07	4,69E-05	1,95E-06	0,0584	8,71E-07		0,1749
**P53**	2,20E-01	0,1509	0,1863	0,1751	0,2221	0,1440	0,3831	0,1744	0,1749	
